# Medial Meniscus Allograft Transplant: Arthroscopic Soft Tissue Technique With Suture Tape Augmentation

**DOI:** 10.1002/atn2.70157

**Published:** 2026-06-29

**Authors:** Sara N. Kiani, Sergio E. Flores, Elly LaRoque, Nicholas Colyvas

**Affiliations:** ^1^ Department of Orthopedic Surgery University of California San Francisco San Francisco California U.S.A.

## Abstract

Meniscal allograft transplant (MAT) is gaining popularity for treating meniscus deficiency. Various MAT techniques are described. Bony techniques such as bone plugs and bone slot techniques are considerably more technically challenging but show better reduction of extrusion than all‐soft‐tissue technique. This MAT technique describes an arthroscopic all‐soft‐tissue fixation, using suture tape augmentation. It combines the technically simpler all‐soft‐tissue MAT method with suture tape augmentation to mitigate hoop stress and act as an ‘internal brace’, thereby adding additional resistance to extrusion. By relying on circumferential suture tape augmentation rather than a centralization stitch to reduce extrusion, this technique more closely replicates the biomechanics of the native meniscus.

## Graphical abstract

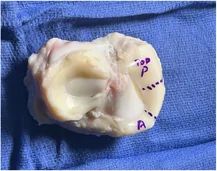

**VIDEO 1:** This meniscal allograft transplant technique describes an arthroscopic all‐soft‐tissue fixation, using suture tape augmentation. It combines the technically simpler all‐soft‐tissue meniscal allograft transplant method with suture tape augmentation to mitigate hoop stress and act as an “internal brace.” Video content can be viewed at https://doi.org/10.1002/atn2.70157.

Meniscus allograft transplant (MAT) is rising in popularity for treatment of medial meniscus deficiency. Results from MAT are generally favorable with a reported 75% to 90% of patients reporting fair to excellent clinical outcomes, with good short‐term, mid‐term, and long‐term improvements, but failure rates are about 30% at 10 years and 40% at 15 years.^1^, ^2^ There is still a lot of variation among surgeons in terms of indications, graft choice, preparation, and fixation methods. The most common fixation methods are all‐soft‐tissue fixation, or bony fixation (bone plug or bony slot fixation). Graft extrusion is thought to have a significant impact on survivability and the chondroprotective ability of MAT because of the reduction in resistance to hoop stresses. Bony techniques are significantly more demanding technically than all‐soft tissue techniques, though biomechanical and radiographic data show improved tibiofemoral contact pressures and less extrusion when the grafts are secured with bone.^3^, ^4^, ^5^, ^6^ However, several clinical studies show no difference in outcomes.^3^, ^7^, ^8^

This MAT technique describes an arthroscopic, all‐soft‐tissue fixation with use of suture tape augmentation to help prevent meniscus extrusion, replicating the biomechanics of a native meniscus. By combining the technically simpler all‐soft‐tissue MAT technique with suture tape augmentation, we aim to provide a method whereby the surgeon can obtain the biomechanical outcomes of the bony techniques, without the considerable technical hurdles of that technique. In addition, the technique we describe does not utilize a centralization suture that other described methods do, thereby better maintaining and replicating the normal mobility of a native meniscus and avoiding the additional incisions and tunnels or anchors required by centralization.

## SURGICAL TECHNIQUE

### Arthroscopic Soft Tissue Technique With Suture Tape Augmentation

Consent was obtained for the images and videos used in this article. After indicating the patient for surgery, the patient was matched to a fresh frozen allograft of a medial tibial plateau with meniscus. This was obtained from the *Musculoskeletal Transplant Foundation (MTF) Biologics* (Edison, New Jersey), which states autonomous consent free from coercion was obtained from the donor(s) or their next of kin; and that tissues were not sourced from executed prisoners or prisoners of conscience.

A narrated description of this technique is available in Video 1. Pearls and Pitfalls of this technique are available in Table 1.

1. Diagnostic arthroscopy, confirmation of meniscus condition and deficiency, evaluation of associated pathologies

**TABLE 1:** Pearls and Pitfalls of Arthroscopic Soft Tissue Fixation With Suture Tape Augmentation for Meniscus Allograft Transplant

Pearls	Pitfalls
• Adequate fat pad debridement should be performed for excellent visualization and easy suture passage. • The surgeon should create adequate portals or consider cannula use to avoid tissue bridges and improve suture management; a suture retriever can be used to run (untangle) sutures before shuttling. • An MCL pie‐crusting technique should be strongly considered. It allows improved visualization and easier passage of instruments, reducing the risk of iatrogenic articular damage and improving repair constructs. • Underlying cartilage and subchondral bone at the footprint should be removed with a curette to provide a good bleeding bed “landing zone” for the meniscus while encouraging mesenchymal stem cell migration. • Before shuttling any sutures through the tunnel, the surgeon should ensure there is no soft-tissue bridge. • The use of a guide pin prior to a reamer ensures confirmation of adequate tunnel placement. • The 4.5‐mm cannulated reamer creates a low‐profile tunnel, which minimizes bone loss and morbidity. Being cannulated, it also allows easy passage of a shuttle suture. • If it is difficult to obtain the correct trajectory for the root tunnel, the surgeon should consider an accessory AM portal or the AL portal as the working portal. • Enlarge your portal to easily accept the girth of the graft to insure no hangup during delivery. • Check skin in meniscus body area with all‐inside anchor deployment and consider adjusting set penetration depth. • Combined sequential small pulls on the root and posteromedial shuttle sutures usually results in the smoothest delivery. • The backup posterior root anchor reliably stabilizes the construct while holding tension. • For anterior root fixation place the knee at 90° and maximize the insertion angle to ensure the anchor goes in at 45°.	• The technique may present a learning curve to surgeons given the use of multiple meniscus repair techniques. • Multiple intra‐articular sutures can increase the risk of a soft‐tissue bridge or suture entanglement. • Failure to inspect the skin after deploying the all‐inside devices could theoretically result in the device anchor deploying outside of the capsule, residing on the skin.

AL, anterolateral; AM, anteromedial; MCL, medial collateral ligament.

The patient receives standard antibiotics and is positioned supine with a thigh post to obtain adequate valgus to access the medial compartment. An exam under anesthesia and a diagnostic arthroscopy is performed with a lateral viewing portal. The medial portal is established to ensure adequate trajectory to the posterior root. If needed, a medial collateral ligament trephination is performed with a spinal needle either on the tibial or femoral attachment, to stretch the medial collateral ligament and allow improved visualization and working room in the medial compartment without increased risk of complications.^9^, ^10^, ^11^ The remaining medial meniscus rim tissue is removed with a shaver.

2. Back table preparation of the graft with root sutures and suture tape augmentation

**FIGURE 1:**
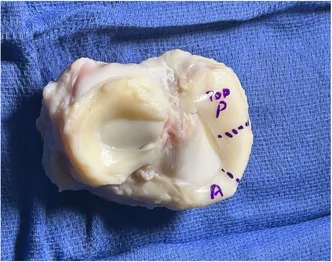
The meniscal allograft preparation. The graft is initially marked with anterior and posterior annotations to insure correct placement in the knee.

**FIGURE 2:**
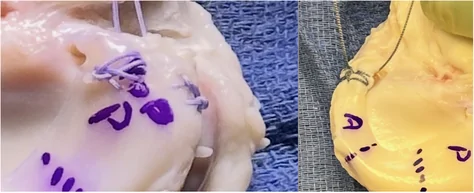
Whip (or Krakow type) suturing of number 2 nonabsorbable suture in both the anterior and posterior horns of the meniscus.

**FIGURE 3:**
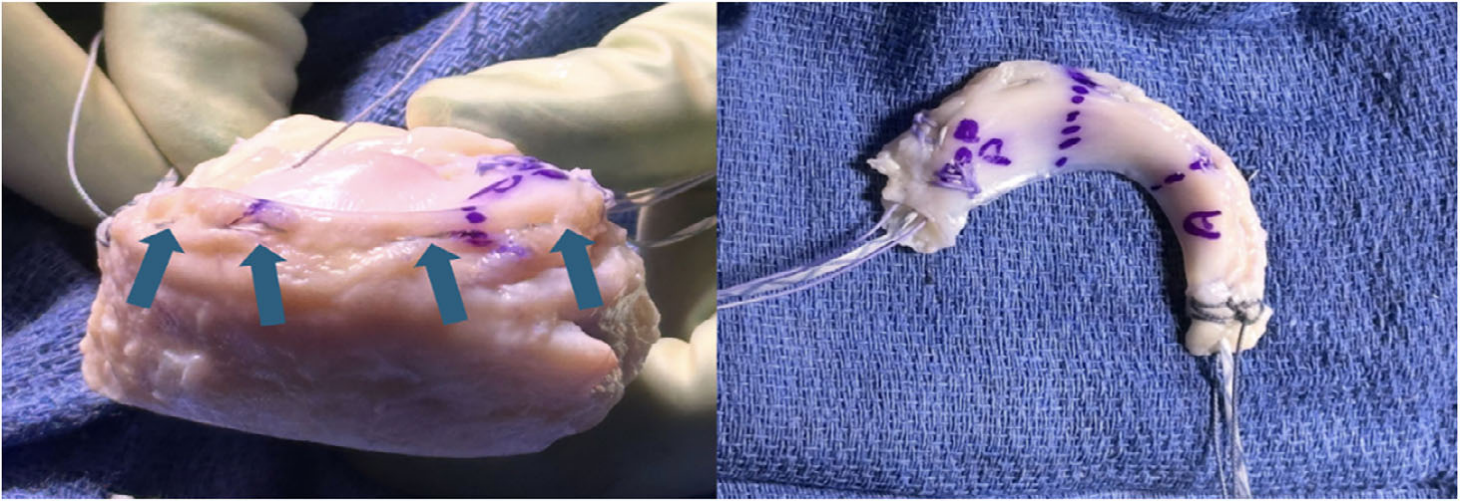
Final graft construct with a circumferential suturing (arrows) of suture tape is performed with the tape ends exiting the anterior and posterior horns.

The medial meniscus allograft is prepared on the back table. First, the anterior and posterior aspect of the meniscus is marked with lines separating the meniscus into thirds (Figure 1). A #2 FiberWire suture (Arthrex, Naples, FL) is secured into the posterior root and anterior root with a Modified Mason‐Allen rip stop stitch (Figure 2). A Krakow stitch may also be used for this step if preferred. A 1.3 FiberTape (Arthrex, Naples, FL) suture is used as suture augmentation on the periphery, starting from the posterior root and exiting from the anterior root (Figure 3).

3. Preparation of the posterior root socket, and passage of shuttle sutures

The posterior root tunnel is drilled with the root guide to the anatomic footprint with a pin and a passing suture (FiberStick, Arthrex, Naples, FL) is used to pass the posterior root sutures from the medial portal through the posterior tunnel. An additional pokehole posteromedial incision is made into the joint and the posterior horn meniscus sutures are shuttled out of the skin to facilitate passing the graft into the joint. A long needle shuttling suture is placed through the Zone Specific II Meniscal Repair System (CONMED, Largo, FL) to pass the stitch in an inside‐out technique.

4. Passage of the graft into the joint, by tension on the root and retraction suture and manipulation with a grasper as needed

**FIGURE 4:**
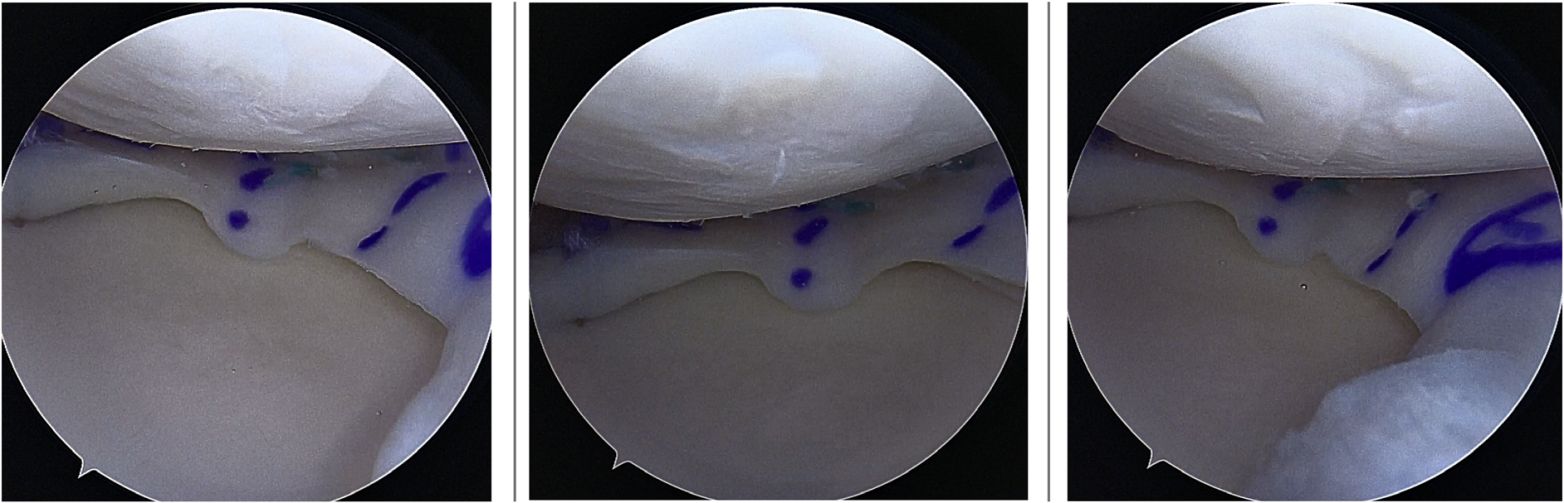
Arthroscopic views from the anterolateral portal of the right medial meniscal transplant in position and sutured in place. A combination of all‐inside, inside‐out, and outside‐in sutures are used.

The meniscus allograft is then transported into the joint through the medial portal. The posterior root suture through the tibial tunnel and the posteromedial passing sutures are then tensioned sequentially to ensure easy delivery and appropriate graft tension.

5. Secure the meniscus to the rim with choice of all inside, inside‐out, and outside‐in suturing techniques

The allograft is then secured with the root and stabilizing sutures and then further secured with a few balanced all Stryker (Portage, MI) AIR inside sutures on the posterior horn and outside‐in sutures on the mid body and anterior meniscus (Figure 4).

6. Secure the anterior horn with suture anchor

The anterior horn is secured with an Argo anchor (CONMED, Largo, FL) or equivalent open or arthroscopically.

### Postoperative Protocol

Postoperative protocol includes nonweight bearing for 4‐6 weeks, range of motion 0‐90 degrees in a hinged knee brace, and physical therapy starting by 2 weeks. At 6 weeks, the patient is made weight bearing and range of motion as tolerated, and the brace is discontinued once the patient has adequate quadriceps control determined by physical therapy. At 6 months the patient can begin running and at 9 months can begin pivoting sports and activities as tolerated.

## DISCUSSION

Using a soft tissue technique for the MAT, the procedure is considerably less technically demanding than bone block/plug technique (Table 2), but biomechanical data suggests higher rates of extrusion with this technique.^3^, ^4^, ^5^, ^6^ However, outcomes appear to be largely the same for both techniques.^3^, ^7^, ^8^ Nonetheless, reduction of extrusion is a worthy goal. The meniscus transplant technique described here aims to recreate the natural biomechanical forces of the meniscus and resistance to extrusion by using circumferential suture tape augmentation. The suture tape augmentation should mitigate extrusion by acting as an “internal brace.” Many techniques advocate for a centralizing stitch through a tunnel in the tibia to prevent extrusion. We believe that this has a high potential to hinder the natural motion and biomechanics of the meniscus and prevent the natural mobility of the meniscus. The suture tape augmentation we describe allows more natural motion to occur and prevents tethering and bunching and “cheesewiring” that can occur with centralization.^12^ Although this article describes the use of this technique for medial meniscus transplants, the senior author has successfully used this technique for both medial and lateral MATs, and it may be particularly beneficial for replicating the normal high level of meniscus mobility on the lateral side. Additional advantages of this procedure include a simpler and easier set of steps to completion, with shorter surgical time. Although centralization is often recommended to prevent extrusion, there is no data showing a clinically important difference and there is concern that the technique may be over‐constraining an area of the meniscus that is not typically constrained.^13^ Furthermore, there could be a higher cost associated with the addition of suture tape and all inside implants, but this is technique dependent. Although we used FiberWire and FiberTape from Arthrex in this procedure, comparable suture and suture tape can be used.

**TABLE 2:** Advantages and Disadvantages of Arthroscopic Soft Tissue Fixation With Suture Tape Augmentation for Meniscus Allograft Transplant

Advantage	Disadvantages
• Shorter and less complicated surgery without the use of bone plugs • Shorter and less complicated surgery without the use of a centralization stitch, which can require an additional tunnel or anchor • Suture tape may mitigate hoop stress and prevent extrusion • More anterior to posterior motion without a centralization stitch may better mimic anatomic meniscus properties • No large medial incision with the use of all inside sutures for the posterior horn	• Lack of a centralization stitch may increase the rate of extrusion • Cost of suture tape and all inside implants increases cost of the procedure

Overall, we feel that any technique modifications that simplify the challenging MAT surgery will be a welcome development. There is increasing evidence that earlier intervention in meniscal deficient knees is of growing value and simplifying the procedure without sacrificing biomechanical integrity will further encourage adoption of MAT.^14^, ^15^ Future research is needed to assess the biomechanical and anatomic effects of suture tape augmentation in meniscus allograft transplant and the ultimate outcomes of these techniques.

## DISCLOSURES

The authors (S.N.K., S.E.F., E.L., N.C.) declare that they have no known competing financial interests or personal relationships that could have appeared to influence the work reported in this article.
